# A soy protein Lunasin can ameliorate amyloid-beta 42 mediated neurodegeneration in *Drosophila* eye

**DOI:** 10.1038/s41598-018-31787-7

**Published:** 2018-09-10

**Authors:** Ankita Sarkar, Neha Gogia, Neil Glenn, Aditi Singh, Gillian Jones, Nathan Powers, Ajay Srivastava, Madhuri Kango-Singh, Amit Singh

**Affiliations:** 1 Department of Biology, University of Dayton, Dayton, OH 45469 USA; 2 Premedical Program, University of Dayton, Dayton, OH 45469 USA; 3 Center for Tissue Regeneration and Engineering at Dayton (TREND), University of Dayton, Dayton, OH 45469 USA; 4 The Integrative Science and Engineering Center, University of Dayton, Dayton, OH 45469 USA; 5 Center for Genomic Advocacy (TCGA), Indiana State University, Terre Haute, IN USA; 6 Department of Biology and Biotechnology Center, Western Kentucky University, 1906 College Heights Boulevard, TCCW 351, Bowling Green, KY 42101 USA

## Abstract

Alzheimer’s disease (AD), a fatal progressive neurodegenerative disorder, also results from accumulation of amyloid-beta 42 (Aβ42) plaques. These Aβ42 plaques trigger oxidative stress, abnormal signaling, which results in neuronal death by unknown mechanism(s). We misexpress high levels of human Aβ42 in the differentiating retinal neurons of the *Drosophila* eye, which results in the Alzheimer’s like neuropathology. Using our transgenic model, we tested a soy-derived protein Lunasin (Lun) for a possible role in rescuing neurodegeneration in retinal neurons. Lunasin is known to have anti-cancer effect and reduces stress and inflammation. We show that misexpression of Lunasin by transgenic approach can rescue Aβ42 mediated neurodegeneration by blocking cell death in retinal neurons, and results in restoration of axonal targeting from retina to brain. Misexpression of Lunasin downregulates the highly conserved cJun-N-terminal Kinase (JNK) signaling pathway. Activation of JNK signaling can prevent neuroprotective role of Lunasin in Aβ42 mediated neurodegeneration. This neuroprotective function of Lunasin is not dependent on retinal determination gene cascade in the *Drosophila* eye, and is independent of Wingless (Wg) and Decapentaplegic (Dpp) signaling pathways. Furthermore, Lunasin can significantly reduce mortality rate caused by misexpression of human Aβ42 in flies. Our studies identified the novel neuroprotective role of Lunasin peptide, a potential therapeutic agent that can ameliorate Aβ42 mediated neurodegeneration by downregulating JNK signaling.

## Introduction

Alzheimer’s Disease (AD; OMIM: 104300), an irreversible, progressive neurodegenerative disorder, which results in the loss of neurons in the hippocampus and cortex. AD manifests as the loss of memory, cognition functions and eventually results in the death of patient^1–4^. The two major causes for AD are accumulation of amyloid plaques and generation of neurofibrillary tangles (NFTs) due to hyper-phosphorylation of microtubule binding protein Tau^1,4–7^. The amyloid plaques are generated from improper cleavage of amyloid precursor protein (APP), a transmembrane protein, by β- and then γ-secretase enzymes to form a 42 amino-acid long fragment, which is referred to as amyloid-beta 42 (Aβ42)^8^. These Aβ42 fibrils self-assemble into extracellular Aβ42 plaques^2,9^. Generally, APP cleavage results in a forty amino acid long polypeptide (Aβ40). The two extra amino acids in Aβ42 polypeptide makes it hydrophobic, which results in accumulation of the amyloid plaques^4–7,10,11^. These Aβ42 plaques cause membrane defects, disruption of neural networks, trigger aberrant signaling and disrupt normal cellular processes resulting in neurodegeneration. Thus, the current consensus is that Aβ42 conversion and self-assembly into oligomeric forms and plaques is responsible for neuronal death in AD by unknown molecular-genetic mechanism(s)^2–7,10,12^. Various animal models of AD have been developed to understand the molecular-genetic basis of AD^13^ as the genetic machinery is highly conserved across organisms including mouse, *C*. *elegans* and fruit fly^4,13–23^.

*Drosophila melanogaster*, fruit fly, is a highly versatile organism to model human disease^13,14,16,22,23^. The *Drosophila* eye is used extensively to model human neurodegenerative disorders^6,13,15,16,20,24,25^ because the important signaling pathways required for development and differentiation of the fly visual system are highly conserved^26^. Thus, the information generated from the fly model can help understand molecular-genetic underpinning of the human disease^13,14,17,19,27^. *Drosophila*, a holometabolous insect, has a blue print for its adult organs housed inside the larva referred to as the imaginal discs^28,29^. The larval eye-antennal imaginal disc gives rise to the adult compound eye, antenna and head upon differentiation^13,26,30–32^. In the developing *Drosophila* eye, the cell fate specification and differentiation is regulated by a group of genes like *twin of eyeless* (*toy*), *eyeless* (*ey*), *eyegone* (*eyg*), *twin of eyegone* (*toe*), *Optix* (*opt*)*; eyes absent* (*eya*), *sine oculis* (*so*), *dachshund* (*dac*) and *optix* (*opt*)^33–39^, which are called retinal determination (RD) genes^40–45^.

The retinal precursor cells in the eye imaginal disc undergo differentiation to form the photoreceptor neurons in the adult eye^26,46–48^. Eight photoreceptor neurons (PR1-8) and several support cells form a unit eye called as the ommatidium. The axons from the photoreceptors (retinal neurons) fasciculate together to form an axonal bundle, which traverses through the optic stalk and then innervate the different layers of the *Drosophila* brain^49,50^. The axons from photoreceptors (PRs) 1–6 terminate in the lamina whereas PR7-PR8 end in a separate layer of medulla after passing through lamina. In the pupal retina, the excessive cells other than the differentiated cells are eliminated by programmed cell death (PCD)^51^. However, abnormal extracellular signaling due to inappropriate levels of morphogens may trigger cell death in the larval eye imaginal disc^52,53^.

Previous work from our lab showed that evolutionarily conserved Wingless (Wg) and Jun-N terminal kinase (JNK) signaling pathway are tightly regulated to allow differentiation to occur and to prevent premature cell death in the developing fields^54^. Wg, a member of highly conserved Wnt/Wg, is responsible for regulating early growth, restricting eye fate and later Wg plays a role in triggering programmed cell death (PCD)^55,56^ in the pupal retina. Wg also plays a role in developmental cell death during larval eye development^53^. Another highly conserved TGF beta (TGFβ) signaling pathway, referred to as Decapentaplegic (Dpp) signaling in *Drosophila*^57–59^, collaborates with Hedgehog (Hh) signaling to promote retinal differentiation in the developing eye as well as antagonize Wg signaling^60^.

Activation of JNK signaling or stress activated kinase proteins of the mitogen-activated protein kinase (MAPK) superfamily trigger cell death^27,61–63^. JNK signaling is activated through a cascade of phosphorylation by MAP Kinases to regulate cell homeostasis^61,64–66^. JNK signaling acts downstream of the Tumor Necrosis Factor (TNF) homolog Eiger (Egr) and its receptor Wengen (Wgn) by Tak1 (TGF-β- activating kinase 1), a JNK kinase kinase (JNKKK), Hemipterous (Hep); a JNK Kinase, Basket (Bsk; Jun kinase) and Jun^63–65,67^. It is known that activation of JNK signaling leads to induction of cell death to eliminate developmentally aberrant cells^63,67^. The functional read out for the activation of JNK signaling is *puckered* (*puc*), which encodes a dual phosphatase, and acts via a negative feedback loop to downregulate the JNK activity^27,63,66^.

We developed a transgenic model system in *Drosophila* eye where we misexpress high levels of human amyloid-beta (Aβ42) in the differentiating retinal neurons of the developing fly retina^27^ using a Glass Multiple Repeat (GMR) Gal4 driver^68^. The transgenic flies with GMR-Gal4 driven UAS-Aβ42 have been abbreviated as GMR > Aβ42^27^. Targeted misexpression of human Aβ42 (GMR > Aβ42) in the differentiating photoreceptors (retinal neurons) of the developing *Drosophila* eye^69^, exhibit progressive neurodegenerative phenotypes that mimic the neuropathology of AD patients^27^. The frequency of this GMR > Aβ42 phenotype is 100%, which makes this *Drosophila* eye model a highly reliable tool for identifying the genetic modifiers of the GMR > Aβ42 mediated neurodegeneration^27,70,71^. We used our AD model to test plant-based protein Lunasin for its role in blocking Aβ42 mediated neurodegeneration. Lunasin, a soy (glycine max) derived peptide, has multiple roles^72^. Lunasin protein has four functional domains^72–74^. It has an N terminal region of unknown function, followed by a chromatin binding helical region, a carboxy terminal RGD cell adhesion motif, and an eight aspartic acid (poly-D) tail. The poly-D tail and RGD motif have been shown to be essential for the bioactivity of Lunasin. Lunasin is known to have anti-cancer effects^74,75^ and reduces stress and inflammation. The odds of manifestation of AD increases with chronic low-grade inflammation like stress, depression, and obesity^76^. Here we present identification of a plant protein Lunasin that can rescue Aβ42 mediated neurodegeneration. This neuroprotective function of Lunasin is achieved by downregulating JNK signaling dependent cell death in the developing retinal neurons of the *Drosophila* eye. Furthermore, gain-of-function of Lunasin can also reduce the mortality rate of the flies expressing Aβ42 in the nervous system.

## Materials and Methods

### Fly Stocks

All fly stocks used in this study are listed and described in Flybase (http://flybase.bio.indiana.edu). The fly stocks used in this study were Canton-S (Wild-type), GMR-Gal4^68^, elav-Gal4 (BL#485)^77^, UAS-Aβ42^27^, UAS-*puc*, *puc*^*E69*^, where lacZ reporter express under the control of *puc* regulatory element, and acts as the functional read out of JNK signaling pathway^66^. Other stocks used were UAS-*Djun*^*aspv7*^ ^78^, UAS-*hep*^*Act*^, *wg*-lacZ^79^, *dpp*-lacZ^80^, where lacZ reporter^81^ express under the control of *wg* and *dpp* regulatory element. The UAS- Aβ42 transgenic flies were generated by microinjecting a UAS-construct where two tandem copies of human amyloid -β1-42 (Aβ42) fused to signal peptide for secretion were cloned^10,25,27^. The rationale of bi-cistronic construct was to mimic APP duplications associated with early onset of familial AD and to express high levels of Aβ42 to induce strong eye phenotype^25,82^.

### Generation of EGFP-Lunasin Transgenic flies

We employed gene tagging approach^83^ to generate EGFP-Lunasin transgenic flies. The sequence for EGFP (Enhanced Green Fluorescent Protein) was fused to the 5′ end of Lunasin sequence^84^. It has been shown that Lunasin tagged with EGFP show no observable differences in bioactivity^73^. The sequence of EGFP-Lunasin with start, stop codons and the restriction sites was synthesized *in vitro*, sequence verified and cloned into pUAST vector. The GFP reporter can provide spatio-temporal localization of Lunasin transgene. The clones were sequence verified and microinjected in *Drosophila* embryos and the transgenic flies were generated. These flies were balanced and used for genetic crosses.

### Genetic Crosses

We employed a Gal4/UAS system for targeted misexpression studies^69^. All Gal4/UAS crosses were maintained at 18 °C, 25 °C and 29 °C, unless specified, to sample different induction levels. The adult flies were maintained at 25 °C, while the cultures after egg laying (progeny) were transferred to 29 °C for further growth. Misexpression of Aβ42 in the differentiating retina (GMR-Gal4 > UAS-Aβ42) exhibits a stronger neurodegenerative phenotype at 29 °C with no penetrance^27,68^. All the targeted misexpression experiments were conducted using the Glass Multiple Repeat driver line (GMR-Gal4)^68^ or embryonic lethal abnormal visual system (elav-GAL4) line^77^. GMR- Gal4 directs expression of transgenes in the differentiating retinal precursor cells of the developing eye imaginal disc and pupal retina^68^. The elav-Gal4 drives expression in the neurons^77^.

### Immunohistochemistry

Eye-antennal discs from wandering third instar larvae were dissected, and fixed in 4% paraformaldehyde in Phosphate Buffered Saline (PBS), and stained following the protocol^85–87^. The primary antibodies used were rabbit anti-Dlg (1:200; a gift from K. Cho), mouse anti-Wg [1:50,Developmental Studies Hybridoma Bank,(DSHB)], rat anti-Elav (1:50; DSHB), mouse anti-Dlg (1:100; DSHB), mouse anti-22C10 (1:100; DSHB), mouse anti-Chaoptin (MAb24B10) (1:100; DSHB^88^), mouse anti-Ey (1:100, DSHB), mouse anti-Eya (1:100, DSHB), mouse anti-Dac (1:100, DSHB), mouse anti-β-galactosidase (1:100; DSHB), rabbit anti-β-galactosidase (1:200) (Cappel), and mouse anti-GFP (1:100, GFP-G1, DSHB). Secondary antibodies (Jackson Laboratories) used consisted of donkey anti-rabbit IgG conjugated with FITC (1:200), donkey anti-mouse IgG conjugated with Cy3 (1:250), and goat anti-rat IgG conjugated with Cy5 (1:250). The tissues were mounted in Vectashield (Vector labs) and all immunofluorescence images were captured using the Laser Scanning Confocal Microscopy^89^ (Olympus Fluoview 1000). All images were taken at 20X magnification unless stated otherwise. The final images and figures were prepared using Adobe Photoshop CS6 software.

### Detection of Cell Death

Cell death was detected using TUNEL assays^27,53,54,90,91^. TUNEL assays were used to identify the cells undergoing cell death where the cleavage of double and single stranded DNA is labeled by a Fluorescent tag (TMR Red). The fluorescently labeled nucleotides are added to 3′ OH ends in a template-independent manner by Terminal Deoxynucleotidyl Transferase (TdT). The fluorescent label tagged fragmented DNA within a dying cell can be detected by fluorescence or confocal microscopy^89^. Eye-antennal discs after secondary antibody staining were blocked in 10% normal donkey serum in phosphate buffered saline with 0.2% Triton X-100 (PBT) and labeled for TUNEL assays using a cell death detection kit from Roche Diagnostics (In Situ Cell Death Detection Kit, TMR red,12156792210).

The TUNEL positive nuclei were counted to determine the dying cell population from five sets of imaginal discs and were used for statistical analysis using Microsoft Excel 2013. The P-values were calculated using two-tailed *t*-test and the error bars represent Standard Deviation from Mean^27,70,71,92^.

### Adult Eye Imaging

Adult flies were prepared for imaging by freezing at −20 °C for approximately 2 hours followed by mounting the fly on a dissection needle^54,86^. The needle with the fly was aligned horizontally over a glass slide using molding putty. Images were captured on an MrC5 color camera mounted on an Axioimager.Z1 Zeiss Apotome using Z-sectioning approach. Final images were generated by compiling the individual stacks from the Z-sectioning approach using the extended depth of focus function of Axiovision software version 4.6.3.

### Western Blot

Protein sample were prepared from third instar eye imaginal disc from Wild type, GMR > Aβ42, GMR > Aβ42 + Lun larvae following the standardized protocol^27,93^. The Phospho SAPK/JNK (Cell Signaling Thr183/Tyr185) (81E11) Rabbit antibody was used at 1:1000 dilution. Signal was detected using Horse Radish Peroxidase (HRP) conjugated goat anti–rabbit IgG using supersignal chemiluminescence substrate (Pierce). Images were captured using the BioSpectrum® 500 Imaging System.

### Eclosure Assay

Eclosure assays are desirable assays to screen the effect of genetic backgrounds on eclosion of flies^77^. We collected eggs on a grape plate from elav-Gal4 (control), elav-Gal4 drive UAS- Aβ42 (elav-Gal4 > Aβ42) and elav-Gal4 drive UAS-Aβ42 + UAS-Lunasin (elav-Gal4 > Ab42 + Lun) flies. The eclosion assay was carried out in four sets of 50 larvae each. We seeded first instar larvae (50 in each set) from a synchronous culture in each vial. We counted 200 larvae (4 sets of 50 larvae) for each cross. The larvae were allowed to develop and hatched/eclosed adults were counted. All unhatched pupae were also counted. All four sets of fifty larvae each for every genotype were seeded from the same grape plate to maintain consistency and accuracy in larval staging.

## Results

### Lunasin can rescue Aβ42 mediated neurodegeneration

**Figure 1:**
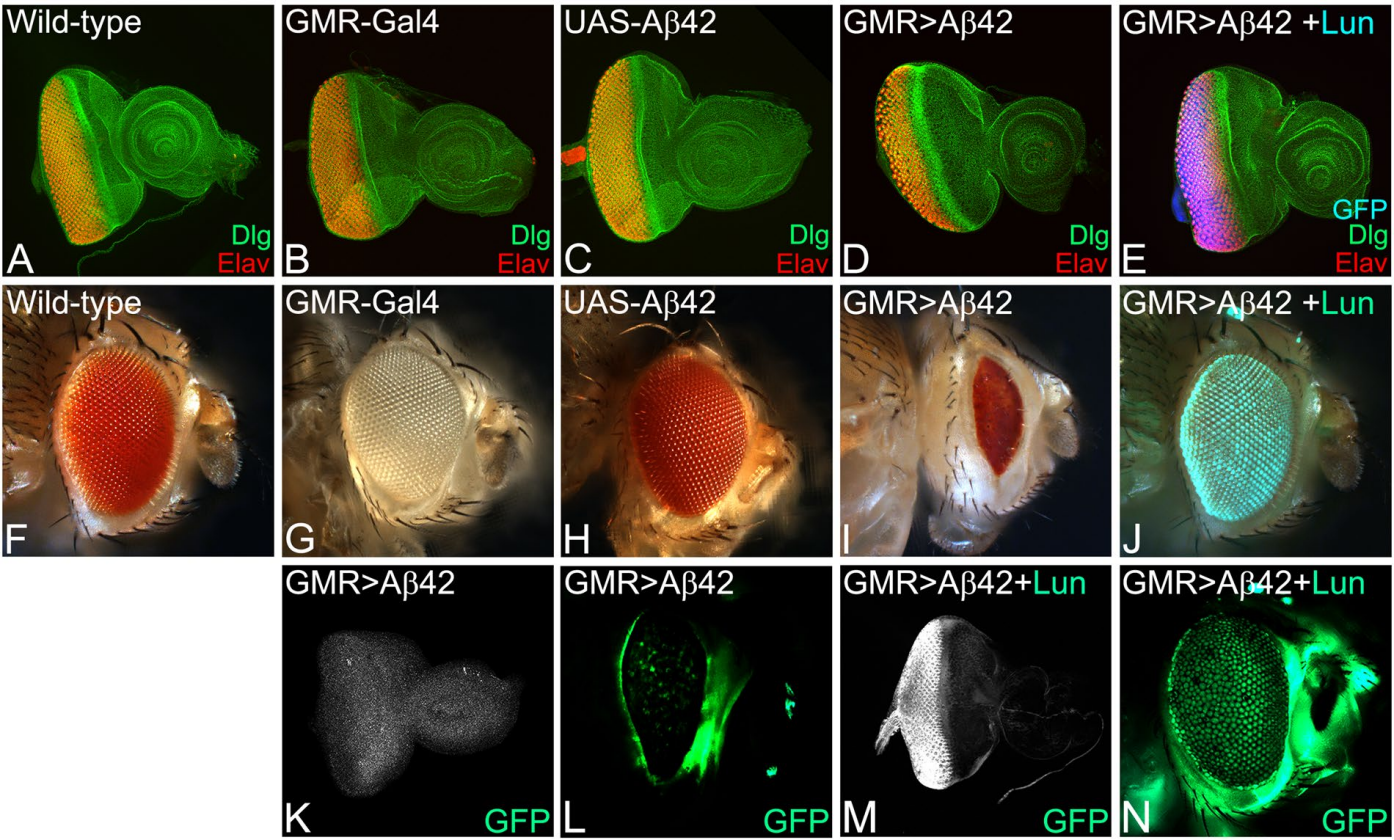
Misexpression of the soy protein Lunasin can rescue Aβ42 mediated neurodegeneration. (**A**,**F**) Wild-type (**A**) larval eye imaginal discs, which develop into (**F**) adult compound eyes comprising of nearly 800 unit eyes. Note that the eye imaginal disc is stained with membrane specific marker Disc large (Dlg: Green) and pan neural marker Embryonic Lethal Abnormal Vision (Elav: Red), which marks nuclei of the retinal neuron. In comparison to the wild-type eyes, (**D**,**I**) misexpression of Aβ42 (GMR > Aβ42) in the differentiating retinal neurons using GMR-Gal4 driver results in the induction of neuronal death as seen in (**D**) eye imaginal discs and the (**I**) highly reduced adult eyes. Note that phenotype worsens from (**D**) larval eye imaginal disc to the (**I**) the adult eye. The controls used are (**B**,**G**) GMR-Gal4 and (**C**,**H**) transgene stock UAS-Aβ42. (**E**,**J**) Misexpression of soy polypeptide Lunasin (Lun) along with Aβ42 (GMR > Aβ42 + Lun) results in significant rescue of Aβ42 mediated neurodegeneration as seen in (**E**) the eye discs and (**J**) the adult eyes. Since Lunasin is a plant protein, in order to determine if Lunasin (Lun) is actually expressed in our model system it has been tagged with EGFP (as seen in **E**,**J**). Note that GFP reporter is detected in GMR domain of (**E**) eye imaginal discs and (**J**) adult eyes. (**K**,**M**) Expression of Lunasin detected by GFP antibody staining in (**K**) control GMR > Aβ42 and (**M**) GMR > Aβ42 + Lun. Note that GFP antibody positively marks the GMR domain only in the (**M**) GMR > Aβ42 + Lun eye disc. In adult eyes, GFP expression is detected by GFP reporter expression in control (**L**) GMR > Aβ42 and (**N**) GMR > Aβ42 + Lun. The orientation of all imaginal discs in the figure is posterior to left and dorsal up. Magnification of all eye discs is 20X.

In comparison to the wild-type eye imaginal discs (Fig. 1A) that develop into adult compound eyes (Fig. 1F), targeted misexpression of Aβ42 in the developing *Drosophila* eye using the GMR-Gal4 enhancer results in a strong neurodegenerative phenotype in the eye imaginal disc (Fig. 1D), and the adult eye (Fig. 1I)^27^. The neurodegenerative phenotype in the GMR > Aβ42 eye imaginal disc (Fig. 1D) worsens in the adult eye (Fig. 1I). The adult eyes are highly reduced with glazed appearance due to fusion of individual unit eyes and also exhibits some dark necrotic spots to mark neurodegeneration (Fig. 1I)^27,70,71,92^. The frequency of GMR > Aβ42 phenotype in the eye imaginal discs (Fig. 1D, n = 72) as well as adult eyes (Fig. 1I, n = 151) is 100%. The controls GMR-Gal4 alone (n = 75, 100%, Fig. 1B,G) and the transgene stock UAS-Aβ42 alone (n = 75, 100%, Fig. 1C,H) exhibits near wild-type eye phenotype in the eye imaginal discs and the adult eyes. The gene tagging with standardized immune-epitopes or fluorescent tags that permit live imaging and do away with the requirement of generating antibodies against a protein is commonly used approach^83^. We employed gene-tagging approach to generate UAS- based transgenic flies where the Soy plant based peptide Lunasin (Lun) is tagged with EGFP (UAS-Lun-EGFP). Misexpression of UAS-Lun-EGFP along with Aβ42 (GMR > Aβ42 + Lun-EGFP) exhibits a strong rescue (n = 100, 70%) resulting in a near complete wild-type eye (Fig. 1E,J). There were no necrotic spots observed in the adult eyes (Fig. 1J). We confirmed that rescue of GMR > Aβ42 neurodegenerative phenotype is due to misexpression of Lunasin based on EGFP transgene expression in the GMR domain of the eye imaginal discs (Fig. 1M) and the adult eyes (Fig. 1N). A strong robust GFP expression was detected by GFP antibody staining in the GMR domain of the GMR > Aβ42 + Lun-EGFP eye imaginal discs (Fig. 1M) and GFP reporter expression was seen in the adult eyes (Fig. 1N). The GFP protein was not detected in GMR > Aβ42 eye imaginal discs (Fig. 1K), and, no GFP reporter expression was seen in the adult eyes (Fig. 1L). We also verified the data using a UAS-Lun construct which is not tagged with EGFP (data not shown). These data suggest that misexpression of Lunasin can rescue GMR > Aβ42 mediated neurodegeneration likely by blocking cell death.

### Lunasin can rescue Aβ42 mediated cell death in *Drosophila* eye

**Figure 2:**
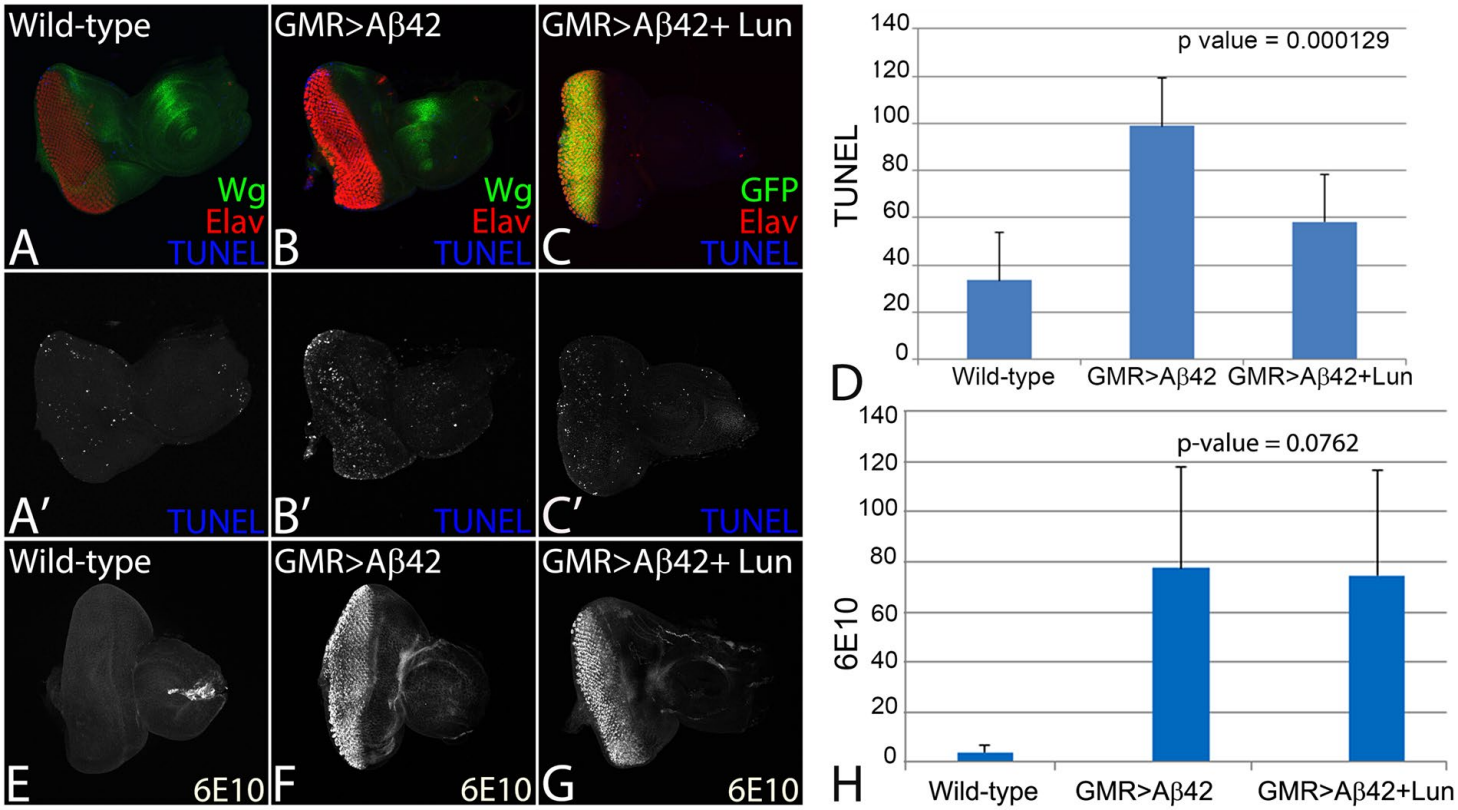
Lunasin can block cell death to rescue Aβ42 mediated neurodegeneration. The dying cells nuclei can be marked by TUNEL staining. TUNEL staining was carried out in (**A**,**A’**) Wild-type, (**B**,**B’**) GMR > Aβ42, (**C**,**C’**) GMR > Aβ42 + Lun eye imaginal discs. The number of dying retinal neurons were counted in these backgrounds (n = 5). Note that the number of dying cells increase nearly 3–4 fold in (**B**,**D**) GMR > Aβ42 as compared to the (**A**,**D**) Wild-type eye discs. (**C**,**D**) Misexpression of Lunasin (Lun) along with GMR > Aβ42 (GMR > Aβ42 + Lun-GFP) results in significant reduction in the dying retinal neurons. The number of TUNEL positive nuclei were counted from five eye imaginal discs for all three backgrounds. (**D**) A graph comparing the number of dying nuclei of neurons validate that Lun misexpression along with GMR > Aβ42 (GMR > Aβ42 + Lun-GFP) rescues the GMR > Aβ42 neurodegeneration. These numbers are significant based on the calculations of P-values using the two-tailed t- test using Microsoft Excel 2013. (**E**–**H**) Accumulation of amyloid plaque was detected using monoclonal antibody 6E10 in (**E**) Wild-type, (**F**) GMR > Aβ42 and (**G**) GMR > Aβ42 + Lun eye imaginal discs. (**H**) The signal intensity of 6E10 staining was calculated from five (n = 5) eye discs of each background and plotted on a graph. Note that 6E10 levels are not significantly different between (**F**) GMR > Aβ42 and (**G**) GMR > Aβ42 + Lun background. The levels of amyloid plaques are barely detected in Wild-type background. Magnification of all eye disc is 20X.

To test this, we employed the TUNEL staining, which marks the dying cell nuclei by labelling the 5′ end of the double and single stranded DNA with a fluorochrome. The fluorochrome tagged TUNEL positive nuclei can be counted to quantify cell death^27,90,91,94^. We performed TUNEL staining in the third instar eye-imaginal disc in the wild-type (Fig. 2A,A’), GMR > Aβ42 (Fig. 2B,B’) and GMR > Aβ42 + Lun (Fig. 2C,C’) backgrounds. The TUNEL staining was performed before the onset of developmentally controlled programmed cell death. The TUNEL positive cells were counted from five sets of imaginal discs from all three backgrounds, and the P values were calculated^27,70,71,92^. A quantification (n = 5, p < 0.05) of dying cells from these genotypes further confirms that in comparison to the wild-type eye disc, a 3–4 fold increase in cell death was observed in GMR > Aβ42 (Fig. 2B,B’,D) background. In comparison to the GMR > Aβ42 (Fig. 2B,B’,D) background, the number of TUNEL positive dying nuclei were significantly reduced in GMR > Aβ42 + Lun (Fig. 2C,C’,D). It suggests that expression of Lunasin is capable of inhibiting cell death as seen by the strong rescue phenotype in the adult eyes.

We wanted to test if Lunasin can rescue GMR > Aβ42 phenotype by preventing accumulation of amyloid plaques or act downstream of amyloid plaque formation in the GMR > Aβ42 + Lun background. Using 6E10 antibody to mark Aβ42 plaques, we found that there is a little or no Aβ42 present in the wild-type (Fig. 2E,H, n = 50, 100%) eye discs. However, there is strong deposition of Aβ42 plaques in GMR > Aβ42 (Fig. 2F,H, n = 52, 100%) and GMR > Aβ42 + Lun (Fig. 2G,H, n = 46, 100%) eye discs. Furthermore, there is no significant difference between Aβ42 plaque formation in GMR > Aβ42 and GMR > Aβ42 + Lun (Fig. 2H) background. It suggests that Lunasin misexpression does not affect Aβ42 plaques formation and acts downstream to GMR > Aβ42 plaque formation.

### Lunasin can restore axonal targeting defects seen in Aβ42 background

**Figure 3:**
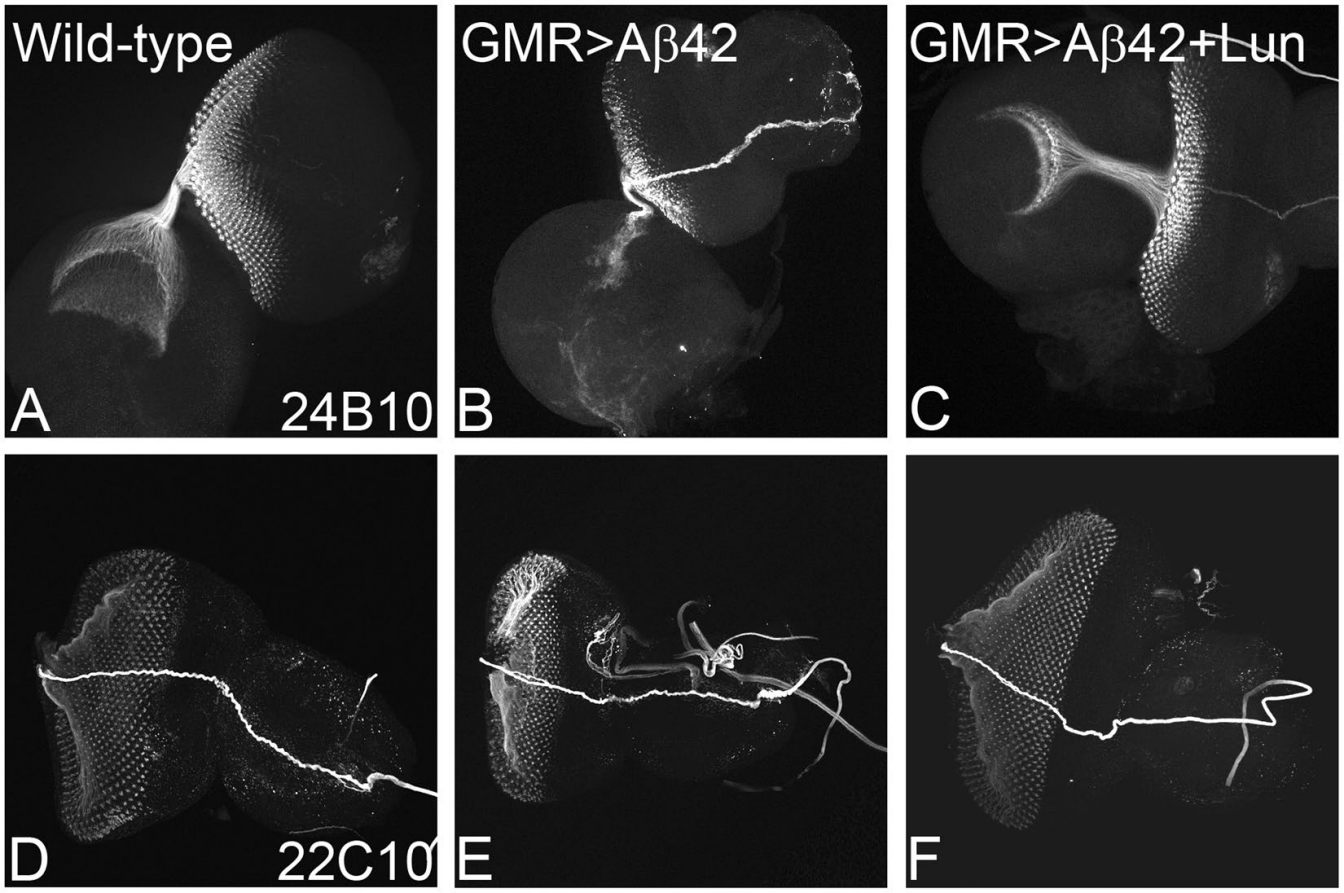
Lunasin (Lun) misexpression can restore Aβ42 mediated impairment of axonal targeting from retina to brain. Chaoptin (MAb24B10), a marker for the axonal targeting from retina to the optic lobes of the brain. (**A**) In wild-type eye imaginal discs, the retinal axons marked by MAb24B10 innervate the lamina and medulla of the brain. (**B**) Misexpression of GMR > Aβ42 in the developing eye imaginal discs result in impaired targeting of retinal axons to the brain. However, (**C**) misexpression of Lunasin (Lun) along with Aβ42 (GMR > Aβ42 + Lun) resulted in significant restoration of the axonal targeting to near wild-type. Monoclonal antibody 22C10 marks all axonal sheath of the photoreceptors in the developing eye. 22C10 expression in (**D**) Wild-type, (**E**) GMR > Aβ42 and (**F**) GMR > Aβ42 + Lun background. Note that impairment of 22C10 expression in GMR > Aβ42 background is restored significantly in GMR > Aβ42 + Lun background. Magnification of all eye discs is 20X.

The neurodegenerative phenotype in AD encompasses disruption of axonal transport mechanism, which results in impaired axonal targeting (incorrect axonal guidance)^27,70,71,92^. In order to understand, if these GMR > Aβ42 + Lun imaginal discs where neurodegeneration phenotype is rescued have proper connection between retinal neurons and brain, we employed 24B10 (Chaoptin, which marks photoreceptor neurons and their axons)^88^. In the wild-type eye disc, R1-R6 axons of each ommatidium project to the lamina whereas R7 and R8 axons project to the medulla, a separate layer of the optic lobe^95^ (Fig. 3A) in (n = 51)100% eye imaginal discs. In comparison to the wild-type eye imaginal discs, the GMR > Aβ42 eye discs show a severe disorganization in axonal targeting (Fig. 3B) in (n = 47)100% larval eye imaginal discs. However, misexpression of Lunasin in GMR > Aβ42 (GMR > Aβ42 + Lun) background significantly restore the axonal targeting (Fig. 3C) in (n = 50) 70% of larval eye imaginal discs. We also investigated the neurons and their axonal processes using 22C10 marker^96^. In comparison to the wild-type expression of 22C10, that marks the retinal neurons and their processes (Fig. 3D, n = 50,100% eye discs), GMR > Aβ42 background show strong neurodegenerative phenotype (reduced neurons and abnormality in their processes) (Fig. 3E, n = 50, 100% eye discs). However, misexpression of Lunasin in GMR > Aβ42 (GMR > Aβ42 + Lun) background significantly restore the neurodegenerative phenotype (Fig. 3F, n = 50, 80%) as compared to the GMR > Aβ42 (Fig. 3E, n = 50,100%) eye discs.

### Neuroprotective function of Lunasin is independent of retinal differentiation genes

**Figure 4:**
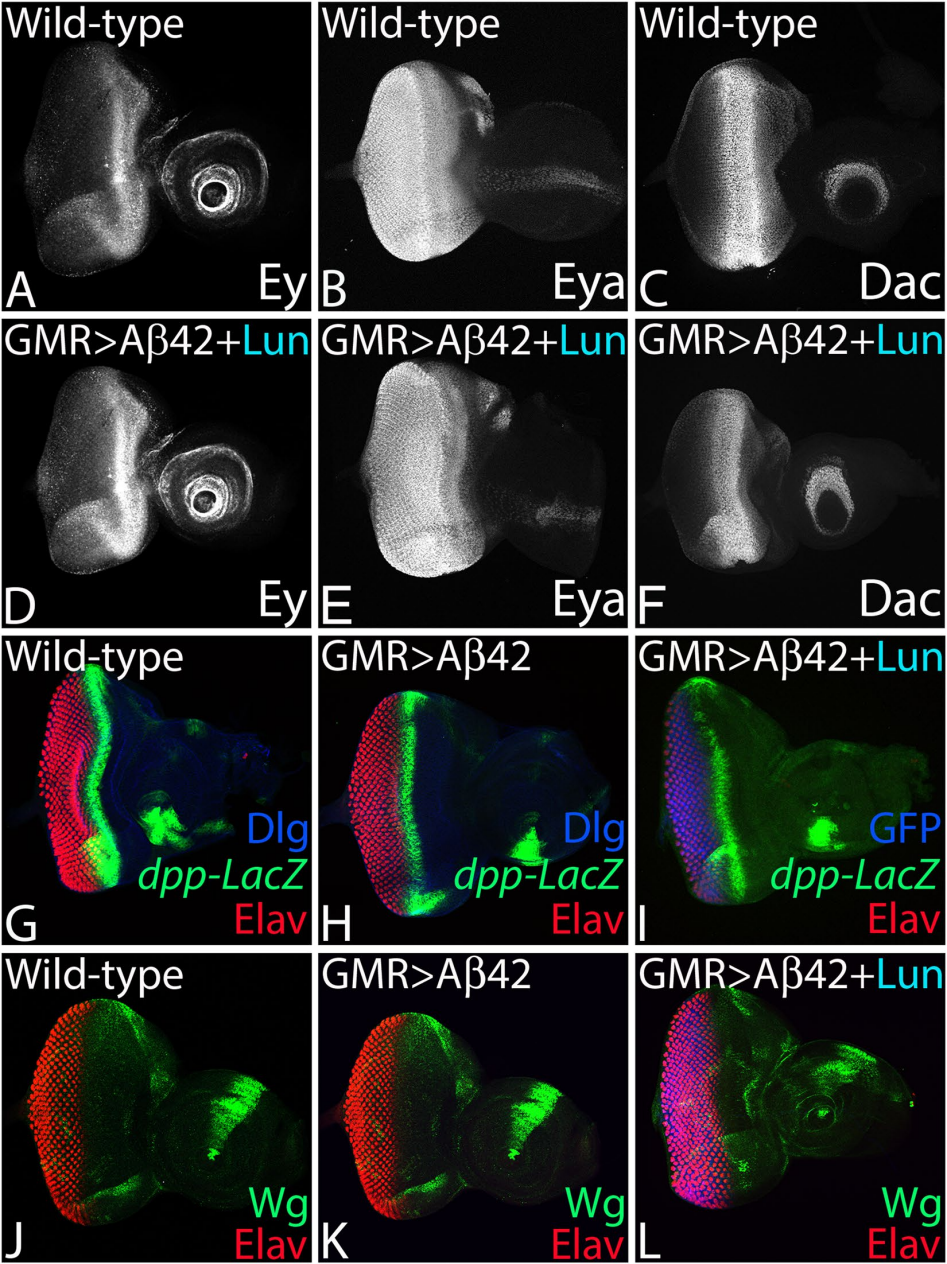
Lunasin neuroprotective function is independent of retinal differentiation gene machinery, Dpp and Wg signaling pathways. (**A**–**C**) Wild-type expression of (**A**) Eyeless (Ey), (**B**) Eyes absent (Eya) and (**C**) Dachshund (Dac), the members of RD gene machinery in developing third instar eye imaginal discs. (**A**) Ey expression is anterior to MF in the third instar eye discs. (**B**) Eya is expressed in the differentiating photoreceptors and anterior to MF. (**C**) Dac is expressed along the MF as well as in antennal region. (**D**) Ey, (**E**) Eya and (**F**) Dac expression is not affected in the GMR > Aβ42 + Lun background. (**G**–**I**) In the developing eye imaginal discs, study of *dpp* expression using *dpp*-lacZ reporter in the (**G**) Wild-type, (**H**) GMR > Aβ42, (**I**) GMR > Aβ42 + Lun. Note that *dpp*-lacZ marks the morphogenetic furrow (MF) in the developing eye. (**J**–**L**) In the developing eye imaginal discs, study of Wg expression using a *wg*-lacZ reporter in the (**J**) Wild-type, (**K**) GMR > Aβ42, (**L**) GMR > Aβ42 + Lun. The *wg* is expressed on the antero-lateral margin of the developing third instar eye imaginal discs. Note that the Lun neuroprotection function is independent of Wg and Dpp signaling. Magnification of all eye discs is 20X.

Since our experimental system is *Drosophila* eye, we need to rule out the possibility of Lunasin affecting the retinal differentiation gene machinery rather than exhibiting the neuroprotective function. We investigated the role of RD genes in neuroprotective function of Lunasin. The Pax-6 homolog *eyeless* (*ey*), eye fate selector gene, is expressed in the early eye and later its expression is restricted anterior to the MF in the eye imaginal disc (Fig. 4A, n = 50, 100%)^30,44,85,97,98^. A tyrosine phosphatase, *eyes absent* (*eya*), is expressed both in the differentiated retinal neurons as well as the retinal precursor cells anterior to MF^30,31,33,70^ (Fig. 4B, n = 50, 100%). Another RD gene *dachshund* (*dac*) is expressed in two different domains one anterior to the MF and another posterior to the MF^70,99^ (Fig. 4C, n = 50, 100%). In GMR > Aβ42 + Lun background the expression of all three RD genes Ey (n = 50, 100%), Eya (n = 50, 100%) and Dac (n = 50, 100%) was not affected (Fig. 4D–F, n = 50, 100%). Our data strongly suggests that the neuroprotective role of Lunasin is independent of RD gene function. In our transgenic model, the Aβ42 expression is triggered at the time of retinal differentiation using a GMR-Gal4 driver^27,68^, which drives expression of UAS- transgene much later than the event of eye specification, and the onset of retinal determination and differentiation genes expression. Thus, neuroprotective function of Lunasin with respect to Aβ42 mediated neurodegeneration is independent of eye specification function or retinal differentiation machinery.

### Lunasin rescues Aβ42 mediated neurodegeneration independent of Wingless (Wg) or Decapentaplegic (Dpp) signaling

Previous studies from our lab and others have shown that accumulation of Aβ42 plaques (GMR > Aβ42) triggers aberrant signaling which results in neurodegeneration^4,27,70,71,92^. We therefore tested (i) several signaling pathways to discern the mechanism of Lunasin mediated neuroprotective function, and (ii) known genetic modifiers of Aβ42 mediated neurodegeneration^27,70,71,92^ (data not shown). The evolutionarily conserved Wg signaling act antagonistically to Dpp during eye development^60^. Wg acts as the negative regulator of eye fate. Gain-of-function of *wg* can suppress the eye fate and loss of function of *wg* induces eye enlargements^44,58,59,85^. In the developing *Drosophila* eye, Wg functions antagonistically to Dpp that promote cell survival. Dpp is also involved in retinal differentiation^60^. During eye development, Wg is expressed on the antero-dorso-ventral eye margin (Fig. 4J, n = 50, 100%) where as Dpp is expressed dynamically along with the MF (Fig. 4G, n = 50, 100%). We found that Lunasin misexpression in GMR > Aβ42 (GMR > Aβ42 + Lun) background does not affect Wg (Fig. 4L, n = 50, 100%) and/or Dpp (Fig. 4I, n = 50, 100%) expression in the eye imaginal discs. Thus, neuroprotective function of Lunasin with respect to Aβ42 mediated neurodegeneration is independent of Wg and Dpp Signaling pathways.

### Lunasin downregulates JNK signaling to prevent neurodegeneration

**Figure 5:**
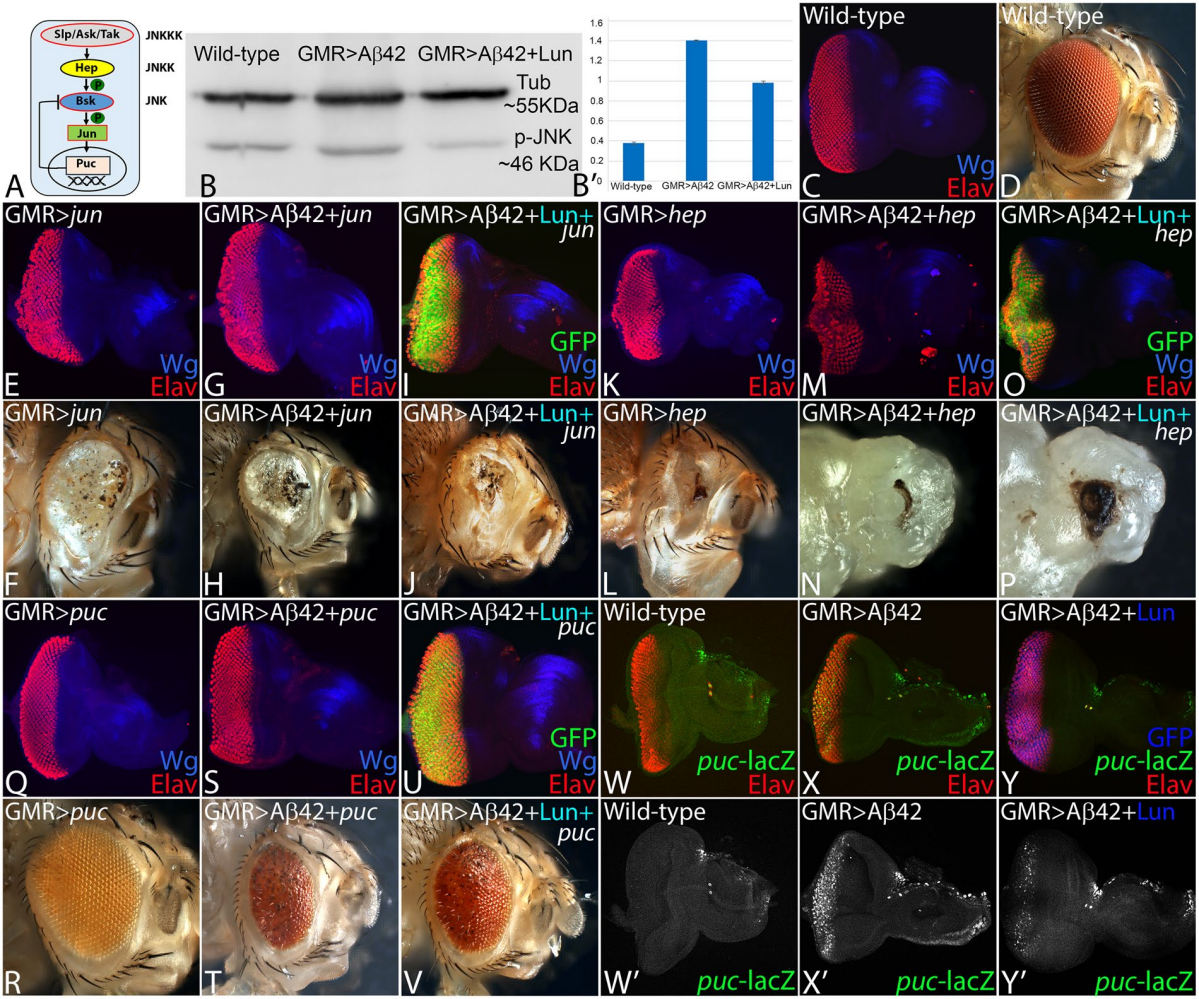
Lunasin downregulates JNK signaling to block Aβ42 mediated neurodegeneration. (**A**) Schematic presentation of JNK signaling pathway. (**B**) Levels of phospho-JNK (pJNK) in a semi-quantitative Western Blot can provide the status of JNK signaling. The higher levels of JNK signaling in GMR > Aβ42 as compared to the wild-type background were significantly downregulated in GMR > Aβ42 + Lun background. The tubulin bands served as controls to normalize the levels of total protein loaded in all three conditions. The p-JNK band staining intensity was calculated by ImageJ. In comparison to the wild-type (**C**) eye imaginal discs and (**D**) adult eyes, activation of JNK signaling in GMR domain using (**E**,**F**) *Djun*^*aspv7*^ (GMR > *jun*) and (**K**,**L**) constitutively active *hep*^*Act*^ (GMR > *hep*) result in strong neurodegenerative phenotype. Furthermore, activation of JNK signaling in GMR > Aβ42 background (**G**,**H**) GMR > Aβ42 + jun (**M**,**N**) GMR > Aβ42 + *hep* exhibits stronger neurodegenerative phenotype which are not rescued by misexpression of Lunasin (**I**,**J**) GMR > Aβ42 + Lun + jun and (**O**,**P**) GMR > Aβ42 + Lun + hep. (**Q**,**R**) Downregulation of JNK signaling by misexpression of *puc*, a dual phosphatase, results in near wild-type (**Q**) eye imaginal discs and (**R**) adult eyes. (**S**–**V**) Misexpression of *puc* in (**S**,**T**) GMR > Aβ42 (GMR > Aβ42 + puc) and (**U**,**V**) GMR > Aβ42 + Lun (GMR > Aβ42 + Lun + puc) results in significant rescue as seen in eye imaginal discs and the adult eyes. The *puc*-lacZ reporter is used as a functional read out of JNK signaling pathway. Expression of *puc-*lacZ reporter (Green) in (**W**,**W’**) Wild-type, (**X**,**X’**) GMR > Aβ42 and (**Y**,**Y’**) GMR > Aβ42 + Lun eye imaginal discs. (**W**,**W’**) Note that *puc* has weak expression in the developing photoreceptor neurons in the wild-type eye imaginal discs. However, (**X**,**X’**) *puc* expression is dramatically upregulated in GMR > Aβ42 background. (**Y**,**Y’**) Misexpression of Lunasin (Lun) along with Aβ42 (GMR > Aβ42 + Lun) can significantly downregulate *puc* expression in the developing third instar eye disc. Magnification of all eye discs is 20X.

We have shown earlier that JNK signaling is involved in Aβ42 mediated neurodegeneration^27^. Activation of JNK signaling triggers a cascade of kinases, which in turn regulates the expression of *puc*^61,63,66,100^. Puc is a dual phosphatase that negatively regulates JNK signaling by a feedback loop^27,63^ (Fig. 5A). The phospho-Jun kinase, encodes an enzyme which can phosphorylate N-terminal its substrate Jun and can be used to study the activation status of JNK signaling. We tested levels of JNK activation by quantifying levels of phospho-JNK in western blots^27^. We quantified and compared the amount of phospho-Jun kinase (p-JNK) in wild-type versus GMR > Aβ42 and GMR > Aβ42 + Lun background. In comparison to the wild-type, p-JNK levels are upregulated in GMR > Aβ42 background as seen earlier^27^. However, in comparison to GMR > Aβ42, pJNK levels were significantly reduced in GMR > Aβ42 + Lun background (Fig. 5B,B’).

We tested if JNK signaling is downregulated by Lunasin (Lun) misexpression. To activate JNK signaling, we misexpressed *Djun*^*aspv7*^ and constitutively active *hemipterous* (*hep*^*Act*^) using the GMR-Gal4 driver and assay its phenotype in the eye imaginal disc and the adult eye (Fig. 5E,F,K,L). In comparison to the wild-type eye (Fig. 5C,D), misexpression of *Djun*^*aspv7*^ alone (*GMR* > *Djun*^*aspv7*^) results in the reduced eye phenotype (Fig. 5E,F, n = 50, 100%). The strong neurodegenerative phenotype of highly reduced eye in GMR > Aβ42 + *Djun*^*aspv7*^ (Fig. 5G,H, n = 50, 100%) was not rescued in GMR > Aβ42 + Lun + *Djun*^*aspv7*^ (Fig. 5I,J, n = 50, 100%) background. Similarly the strong neurodegenerative phenotype due to activation of JNK pathway by using *hep*^*Act*^, as seen in *GMR* > *hep*^*Act*^ (Fig. 5K,L, n = 75, 100%), GMR > Aβ42 + *hep*^*Act*^ (Fig. 5M,N, n = 75, 100%) was not rescued by misexpression of Lunasin (GMR > Aβ42 + Lun + *hep*^*Act*^, Fig. 5O,P, n = 50, 100%). However, blocking or downregulating JNK signaling by misexpression of *puc* in GMR > *puc* exhibits near wild-type eye (Fig. 5Q,R, n = 50, 100%). Misexpression of *puc* in GMR > Aβ42 + *puc*, exhibits significant rescue of GMR > Aβ42 neurodegenerative phenotype (Fig. 5S,T, n = 50, 45%). Furthermore, *puc* expression in GMR > Aβ42 + Lun background (GMR > Aβ42 + Lun + *puc*) can significantly rescue Aβ42 mediated neurodegeneration as seen in the eye imaginal disc and the adult eye (Fig. 5U,V, n = 50, 40%). Thus, Lunasin, which acts upstream of JNK signaling, may downregulate JNK signaling in rescuing Aβ42 mediated neurodegeneration in the *Drosophila* eye.

To test this hypothesis, we analyzed the expression of *puc* (Fig. 5W,W’), a functional read out of JNK signaling pathway by using a *puc*-lacZ reporter^66,101^. There is a robust induction of *puc-*lacZ in GMR > Aβ42 (Fig. 5X,X’, n = 30, 100%) as compared to the wild-type *puc* expression in eye imaginal discs (Fig. 5W,W’, n = 30, 100%). However, in GMR > Aβ42 + Lun, *puc* levels are significantly downregulated (Fig. 5Y,Y’, n = 30, 70%) as compared to GMR > Aβ42 background (Fig. 5X,X’). Our data clearly validate our hypothesis that Lunasin downregulates JNK signaling to rescue Aβ42 mediated neurodegeneration.

### Lunasin increase the mortality of Aβ42 expressing flies

**Figure 6:**
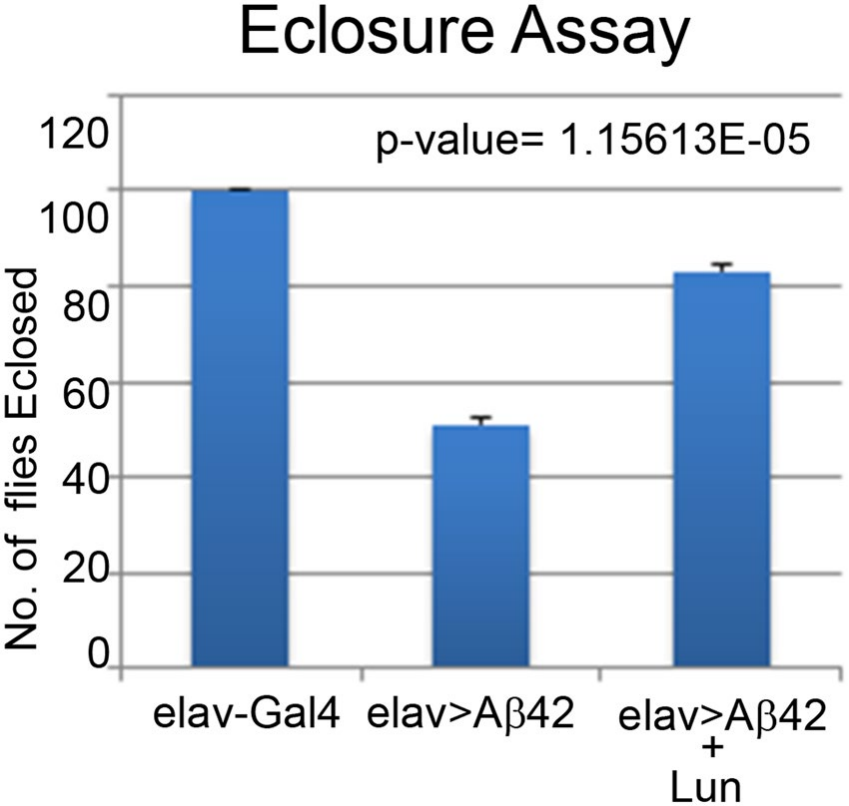
Misexpression of Lunasin can reduce the mortality rate of *elav* > Aβ42 flies. (**A**) A graph comparing the number of flies hatched in Wild-type, *elav* > Aβ42 and *elav* > Aβ42 + Lun background validates that Lunasin misexpression along with *elav* > Aβ42 (*elav* > Aβ42 + LunGFP) rescues the *elav* > Aβ42 mortality rate. We counted 200 flies in three independent sets from each background and plotted on a graph. These numbers are significant based on the calculations of P-values using the two-tailed t- test using Microsoft Excel 2013.

To rule out the possibility that these studies are not restricted only to the retinal neurons, we employed *elav*-Gal4 that drives expression in the neurons of flies^77^. We misexpressed Aβ42 using *elav*-Gal4 (*elav* > Aβ42), which resulted in reduced mortality rate as only 50% (n = 200) of the flies could hatch out and survive whereas remaining 50% population were arrested as larvae or pupae. However, all wild-type flies hatched out and did not show any lethality (Fig. 6, n = 200, 100%). We also analyzed mortality rate when Lunasin is misexpressed in *elav* > Aβ42 (*elav* > Aβ42 + Lun) background. Misexpression of Lun significantly reduced the mortality rate of *elav* > Aβ42 background (Fig. 6, n = 200, 80%). Nearly 80% of the flies hatched out and only 20% flies failed to hatch out due to pupal and larval lethality.

## Discussion

One of the hallmarks of AD is accumulation of amyloid Aβ42 plaques over a period of time, which triggers neuronal death leading to neurodegeneration. This Aβ42 mediated neurodegeneration is an outcome of activation of aberrant signaling because of stress in the neurons^2,4,5,17,27^. Thus, the Aβ42 mediated neurodegenerative phenotype observed in AD is not due to a single gene mutation but an outcome of impairment of several signaling pathways^4,17,19,27,70,71,92^. In order to understand the complexity of this disorder, it is important to identify these signaling pathways.

One of the approaches to discern molecular genetic basis of AD, and to find future cures, it is important to identify the downstream targets of signaling pathways that are triggered as an outcome of the Aβ42 accumulation^4^. The efforts have been directed to search for chemical inhibitors that can block/downregulate these downstream targets of aberrant signaling pathways and thereby prevent/delay Aβ42 mediated neurodegeneration. In this direction, the repertoire of natural product libraries comprising of plant proteins^102,103^ with medicinal properties provide an alternative to the chemical inhibitors which can be screened to identify the ones that can either delay or block the onset of neurodegeneration observed in GMR > Aβ42 background.

Several plant products have been identified as therapeutic targets for cancer, inflammation and various other disease^72,74,75,102,103^. Chronic inflammation has long been implicated in cancer and also plays major role in neurodegenerative disorders like AD. The soy protein Lunasin has multiple interacting domains and may affect different cell biological processes. Lunasin has been reported to have anti-metastatic and chemopreventive activity^61,72,74,75,104,105^. Lunasin can significantly reduce a melanoma stem cell population^106^. It has been suggested that primary anticancer mechanism of Lunasin is based on its activity as a HAT inhibitor^74,107^. HATs have been known to play role in AD.

Our studies demonstrated that Lunasin can rescue Aβ42 mediated neurodegeneration in the *Drosophila* eye (Fig. 1). Lunasin is known to prevent cancer but its role in neurodegenerative disorders have not been tested to date. We tested the possibility if Lunasin is preventing accumulation of amyloid plaques and thereby preventing Aβ42 mediated neurodegeneration. We checked Aβ42 plaque accumulation using monoclonal antibody 6E10 in wild-type, GMR > Aβ42 and GMR > Aβ42 + Lun backgrounds. We found that Aβ42 plaque accumulation was comparable between GMR > Aβ42 and GMR > Aβ42 + Lun backgrounds (Fig. 2). The fact that there is no significant difference in the plaque deposition with or without Lunasin in GMR > Aβ42 background, proves that Lunasin modulates Aβ42 toxicity indirectly, and is downstream of Aβ42 plaque accumulation.

In order to identify and characterize the mechanism behind the novel neuroprotective function of Lunasin, we tested various genetic modifiers of Aβ42 like C2H2 zinc finger transcription factor, Teashirt (Tsh), CREB binding protein (CBP) and apical basal polarity marker, Crumbs (Crb)^27,70,71,92^. We found that the neuroprotective function of Lunasin is independent of Tsh, CBP and Crb (data not shown). Interestingly, among various other functions, CBP acts as a histone acetyl transferase (HAT)^92^, and it is known that Lunasin functions as inhibitor of HAT^107^. However, we did not see any interaction between Lunasin and CBP in GMR > Aβ42 background (data not shown).

**Figure 7:**
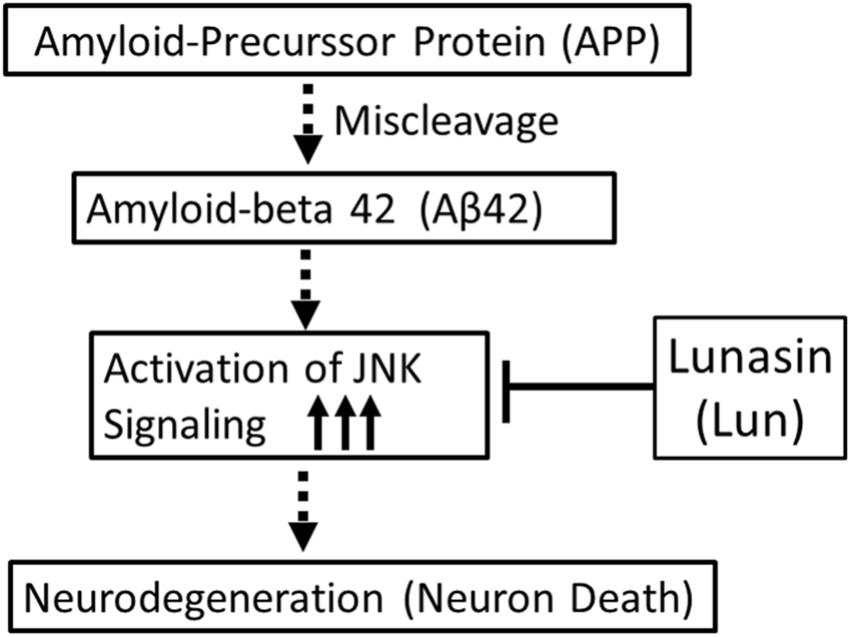
A model to show the mechanism by Lunasin (Lun) blocks Aβ42 mediated neurodegeneration. Accumulation of Aβ42 plaques trigger a cascade of events, which activates JNK signaling. Activation of JNK signaling in the neuron triggers cell death. Misexpression of Lunasin blocks JNK signaling to promote neuroprotection of retinal neurons in the *Drosophila* eye.

In order to discern molecular genetic basis of neuroprotective function of Lunasin, we tested various signaling pathways and found that it is independent of Wg and Dpp signaling (Fig. 4). Finally, we found that neuroprotective function of Lunasin is mediated through downregulation of highly conserved JNK signaling pathway (Figs 5 and 7). Earlier, we have seen that accumulation of Aβ42 plaque causes ectopic induction of JNK signaling pathway in the neurons, which in turn triggers neuronal death^27^. Our data demonstrates that Lunasin misexpression can rescue Aβ42 mediated neurodegeneration by downregulating JNK signaling in the *Drosophila* eye (Fig. 7). Thus, our studies provide evidences for the first time that JNK signaling, an important link in onset, manifestation and progression of AD, can be modulated by plant-based protein. Studies in various animal models of AD suggests the involvement of JNK signaling in AD^108^. Our studies open up new avenues where plant proteins expressed by transgenic approach in the neuron can prevent the onset or delay the onset of AD in the animal model of *Drosophila* eye. Since JNK signaling pathway is known to be involved in developmental processes like ageing, development, tissue homeostasis, cell proliferation, cell survival and innate immune response, the modulation of JNK can be of significance in other disease too like Parkinson, stroke etc^109^.

The results from our present study support the model of potential therapeutic benefits of Lunasin in Alzheimer’s Disease (Fig. 7). Lunasin can prevent progression of Aβ42 mediated neurodegeneration by effectively downregulating JNK signaling. The use of plant-based product can be a promising alternative or addition to the use of gene silencing or directly blocking signaling pathways to treat neurodegenerative disorders. Thus, Lunasin, a major bioactive component of the soy-based food has potential to exert a major impact on human health. Further studies are needed to test the efficacy of Lunasin in other vertebrate model organisms to determine if its anti-inflammatory and JNK inhibitory activities show neuroprotective effects. It will be interesting to see if Lunasin can be developed as a potential natural product for the treatment of Aβ42 mediated neurodegeneration observed in AD.

## Data Availability

The datasets generated during and/or analyzed during the current study are available from the corresponding author on reasonable request.
